# Kinesiophobia in People with Multiple Sclerosis and Its Relationship with Physical Activity, Pain and Acceptance of Disease

**DOI:** 10.3390/medicina58030414

**Published:** 2022-03-10

**Authors:** Dagmara Wasiuk-Zowada, Anna Brzęk, Ewa Krzystanek, Andrzej Knapik

**Affiliations:** 1 Department of Physiotherapy, Faculty of Health Sciences in Katowice, Medical University of Silesia, 40-754 Katowice, Poland; dwasiuk@sum.edu.pl; 2 Department of Neurology, Faculty of Health Sciences in Katowice, Medical University of Silesia, 40-754 Katowice, Poland; ekrzystanek@sum.edu.pl; 3 Department of Adapted Physical Activity and Sport, Faculty of Health Sciences in Katowice, Medical University of Silesia, 40-754 Katowice, Poland; aknapik@sum.edu.pl

**Keywords:** kinesiophobia, multiple sclerosis, physical activity, acceptance of illness scale

## Abstract

*Background and Objectives*: Multiple sclerosis (MS) is the most common chronic demyelinating disease. Factors that reduce the occurrence of symptoms include physical activity (PA). However, the data indicate that PA levels among people with MS are lower than those of healthy peers. The cause may be kinesiophobia. The aim of the study was to determine the level of kinesiophobia among people with MS and its relationship with age, disease duration, functional status, PA, and degree of acceptance of the disease. *Materials and Methods*: Eighty people aged 35–69 were examined: 60 women (75%) and 20 men (25%). The Expanded Disability Status Scale (EDSS) was used to determine the level of disability (median: 3.50; min–max: 1–6). The research questionnaire consisted of a metric section, Visual Analogue Scale (VAS) for pain, Tampa Scale of Kinesiophobia (TSK), Acceptance of Illness Scale (AIS), and Modified Baecke Questionnaire for Older Adults for physical activity. *Results*: Of the respondents, 52.50% were characterized by a high level of kinesiophobia (>37 points). Correlation analysis: TSK and PA showed the following: r = −0.363 (*p* = 0.001). Regression explains kinesiophobia in 44% (R2 = 0.4364; *p* < 0.0000). The predictors of TSK were as follows: disability level: *p* < 0.01, ß = 0.33; disease acceptance: *p* < 0.01, ß = −0.34; PA: *p* < 0.05, ß < −0.05. *Conclusions*: The problem of kinesiophobia is significant in MS patients, and its predictors are the functional status of the patients, low degree of acceptance of the disease, and low level of physical activity. The age and duration of the disease do not determine the problem of fear of movement.

## 1. Introduction

Multiple sclerosis (MS) is the most common chronic demyelinating disease. The first symptoms of MS usually appear in early adulthood and depend on the location of the demyelinating foci [1]. The main problems faced by patients are visual disturbances, sensory disturbances, fatigue, difficulty walking, deficits in motor and cognitive function, and emotional problems [2,3]. The progressive and unpredictable nature of MS causes a significant deterioration in the quality of life of both patients and their relatives [4]. The prevalence of this disease varies widely geographically. The highest rates (>100/100 thousand inhabitants) are in Europe and North America, which suggests, apart from genetic predisposition, the importance of exogenous factors in the etiology of this disease [5,6]. It is estimated that the number of people with MS worldwide reaches 2.3 million [7].

No cure for MS currently exists; however, most cases of MS are treated with disease-modifying therapies (DMTs) [8]. Therefore, scientists and clinicians are looking for effective strategies to minimize symptoms. Among the main factors reducing the occurrence of MS symptoms is physical activity (PA) [9]. Taking up PA by people suffering from MS is associated with a reduction in fatigue and risk of falls, which positively influences the quality of life [10,11,12]. Moreover, the role of PA in shaping balance, coordination, strength, endurance, and gait function among patients with MS is emphasized [13]. There are reports that long-term systematic PA reduces the number of relapses and disease progression in some patients [14]. The explanation for this is the hypothesis that prohealth behaviors, such as taking up PA, support the functioning of the neuroimmune system in order to reduce proinflammatory reactions [15]. The arguments presented here indicate that taking up PA by these patients can be considered one of the best therapeutic strategies for people suffering from MS [16].

Despite the already known beneficial effects of PA on the functioning and quality of life of people with MS, researchers’ reports indicate that its level is generally lower in these patients than in the general population [17,18]. The causes of motor passivity in humans are complex [19], and researchers believe that fear of movement—kinesiophobia—plays a leading role. In chronically ill patients, apart from the typical barriers of the general population, there are also factors related to the type of disease, stage of advancement, and related limitations [15,20,21].

Kinesiophobia in people suffering from neurological diseases is still relatively poorly understood [22]. Few publications on the problem of kinesiophobia in MS patients indicate its association with pain and fatigue and, consequently, its impact on quality of life [23]. In another publication, researchers found a beneficial effect of relaxation techniques on symptoms of pain, fatigue, and kinesiophobia in MS patients [24]. The literature review also found one publication confirming the usefulness of the Tampa Scale of Kinesiophobia (TSK) in the study of kinesiophobia in MS patients [25]. The data cited prove the importance of this problem and the need for research in this direction. Investigating this problem in people suffering from MS and the relationship with age, disease duration, functional status, PA, and the degree of disease acceptance may constitute a premise for the development or modification of patient activation programs.

## 2. Materials and Methods

### 2.1. Participants

The study included 80 people aged 35–69 (mean: 45.51; SD = 8.53). There were 60 women (75%) and 20 men (25%) among the respondents. The selection for the research was deliberate—the participants were people diagnosed with MS. All participants in the study were informed about its purpose and gave their written consent to participate in it. Participant recruitment was conducted among patients treated in a hospital outpatient clinic, a neurological ward of one of the Katowice hospitals (Poland).

Due to the purpose of the study, the following selection criteria were adopted: clinical status according to Expanded Disability Status Scale (EDSS) from 1 (minimal neurological deficits) to 6 (requiring mechanical assistance, such as a cane or walking stick, for ambulation), no features of the dementia syndrome- Mini-Mental State Examination score > 24 points) [26], and the level of disability not greater than 6 points according to the EDSS [27].

### 2.2. Methods

The study was conducted using a questionnaire filled in by the respondents themselves. In case of problems, the examiner explained the ambiguities. The questionnaire consisted of a metric section, where data on sex, age, duration of neurological disease, and its severity were collected. All participants, including the elders, completed the questionnaire themselves. A member of the research team was available throughout the study. Patients could ask questions when they had any doubts.

The level of pain experienced by the participants was measured using the 10-point visual analogue scale (VAS) (0—no pain, 10—unimaginable pain) [28].

The following scales were used in the research:

- The Tampa Scale of Kinesiophobia is used to assess fear of movement. It contains 17 items that are assigned Likert answers (1–4 points). The scoring of Questions 4, 8, 12, and 16 is reversed. The total score is in the range of 17–68 points. The higher the score, the greater the severity of kinesiophobia [29,30].
- The Acceptance of Illness Scale (AIS) measures the degree of acceptance of disease. It consists of eight statements that describe the difficulties and limitations associated with the disease. Each answer is scored in the range 1–5. The final result is in the range of 8–40 points. The following interpretation was adopted: 8–18, low acceptance level; 19–29, medium acceptance level; 30–40, high acceptance level [31,32,33].
- Due to limitations in MS patients, the modified Baecke Questionnaire for Older Adults for physical activity was used to determine the level of PA. The tool is used to estimate the annual level of PA on the basis of the patient’s self-report. Daily activities related to household chores are taken into account—including locomotion, sports, and leisure activities. The intensity of the effort and its duration are appropriately scored. The total PA index is the sum of activities from three appropriately scored areas: household chores, sports activity, and leisure activity [34,35]. The original version of this questionnaire was validated in both the healthy and the sick population [35,36]. In addition, the adaptation of this tool for the purposes of examining the elderly is used by many researchers [37,38,39].

### 2.3. Statistical Analysis

Descriptive statistics were performed. The internal consistency of the TSK was tested by calculating Cronbach’s α. Non-parametric statistics were used for the analyses: relationships between variables—Spearman’s rank correlations; intergroup comparisons—Mann–Whitney U test. The TSK predictors were examined using backward stepwise regression. Level of significance adopted: *p* < 0.05. Calculations were performed in the statistical program Statistica version 12 (StatSoft Polska, Krakow, Poland).

## 3. Results

The preliminary analysis of TSK’s internal consistency showed a satisfactory level. The Cronbach’s α coefficient was 0.77, and the half-time reliability was 0.73. A qualitative assessment of the level of kinesiophobia was also performed. As proposed by Vlayen et al., the result of >37 points was assumed as its high level [40]. There were 38 people below this threshold, which constituted 47.50% of all respondents. In contrast, 42 people (52.50%) had a high level of kinesiophobia.

The descriptive statistics of the studied variables and their comparison by gender (Table 1) did not show any differences. Therefore, the group was treated as homogeneous in further analyses.

In correlation analysis, TSK and PA showed the following relationship: r = −0.363 (*p* = 0.001). The comparison of the PA level of people without kinesiophobia and that of with people with a high level of kinesiophobia showed differences: *p* < 0.05.

The functional status of the patients according to EDSS was correlated with all variables analyzed. Particularly important relationships are noticeable with AIS and TSK (Table 2).

The regression analysis performed, including age, duration of MS, VAS, AIS, and PA as independent variables and TSK as a dependent variable, turned out to be statistically significant. After performing univariate tests and reducing statistically insignificant variables, it turned out that the model explains the determinants of kinesiophobia in 44% (R2 = 0.4364; *p* < 0.0000). The TSK predictors were as follows: EDSS: *p* < 0.01, ß = 0.33; AIS: *p* < 0.01, ß = −0.34; PA: *p* < 0.05, ß < −0.05.

## 4. Discussion

Motor passivity is a well-known predictor of health risks. Kinesiophobia may be a key challenge for therapists in chronically ill patients, especially in those cases where an adequate level of PA improves health or slows disease progression [41,42,43,44,45]. This also applies to selected neurological diseases. This area of disease has so far been poorly researched, and reports are scarce. The presented results, as well as previous studies, indicate that the scale of this problem is large [22,46,47,48,49].

Taking into account the limitation of this study—the number of people tested and its cross-sectional nature—it should be assumed that gender is not a factor influencing the level of kinesiophobia. Functional status analysis (EDSS) shows the relationships of kinesiophobia with age, disease duration, pain intensity, and decline in disease acceptance (AIS). This is consistent with the earlier observations of other researchers [50]. In our study, the correlations are not strong, which, however, seems to confirm the individual course of MS—depending on the type of disease course. The patients’ reactions to the symptoms and limitations associated with MS are also individual. The complexity of these compounds requires further research. This also applies to the PA.

Our research shows that lower PA levels are associated with higher TSK scores, resulting in a “vicious circle”. It is worth adding that people with MS generally have lower levels of PA than their healthy peers [17]. The very awareness of being sick, as well as the actual limitations resulting from the disease, have a great influence on this condition. The type of MS (primary progressive vs. relapsing/remitting) is of great importance [51]. The presented study results indicate that the TSK–PA correlations were relatively low. Some reports indicate that the questionnaire used here for estimating PA is not sensitive. Critical comments apply, in particular to people with an average level of PA [35]. This should be taken into account when interpreting our results. However, we decided that both in the elderly as well as in people with progressive disease leading to disability, the very area of household duties is very important, as the most “basic” activity and necessary for independent functioning. Perhaps a patient-specific PA estimation tool should be considered. This requires further research in this direction.

The analysis of the presented results shows that in people with 4 or more points on the EDSS scale, there is a significant increase in VAS and TSK—with a simultaneous decrease in AIS and TSK scores. These data suggest that this group of patients in particular should be monitored by a therapeutic team consisting of a physician, a physical therapist, and a psychologist in order to minimize the motor symptoms associated with MS. It should be emphasized that the individual course of the disease and its perception also require an individual approach to each patient [52]. An indication may be the experience of researchers with patients after myocardial infarction, where, similarly to MS, exercises and PA are the main components of patients’ rehabilitation. It was found in these studies that kinesiophobia can create a barrier to obtaining the appropriate effects of the rehabilitation process. The quality of the information provided to the patients was important. Inconsistent messages made the fear of movement heightened. Patients experiencing symptoms during activity such as increased heart rate or shortness of breath, combined with excessive vigilance directed at signals from the body, tended to avoid PA in favor of rest and strengthened their akinetic posture. This strengthens the thesis that patients need specific explanations about their condition and prefer individual advice tailored to their needs, rather than universal guidelines for patients [53]. Symptoms in MS patients are different. In addition to typical MS fatigue (primary and secondary) [54], pain [55], and affective disorders [56], Uhthoff’s phenomenon or sensitivity to heat may also be a problematic barrier to activity. It is estimated that about 60–80% of people with MS experience it. An increase in body temperature by up to 0.5 °C can temporarily worsen clinical symptoms. Fear of overheating of the body as a result of improperly conducted physiotherapy or PA (of too high intensity) may, to some extent, explain the attitude of motor passivity in people with MS [57]. Moreover, it should be emphasized that in addition to the aspects related to MS and neurological state, the lack of PA may also have consequences such as hypercholesterolemia, hypertension, type 2 diabetes, cardiovascular diseases [12], and overweight [58]. These diseases can exacerbate the aforementioned “vicious circle” and should be considered when programming activities.

The reports to date seem to confirm the beneficial effect of taking up PA on the emotional and functional state of patients. Intervention in the form of 6-month yoga training in MS patients resulted in a significant improvement in the mental dimension of health-related quality of life, walking speed, fatigue, and depression levels [59]. In other studies, an improvement in the muscle strength of the lower limbs was found during systematic resistance training conducted at home [60]. On the other hand, the intervention in the form of rehabilitation of the balance proved to be effective in reducing the frequency of falls and improving the balance [61]. However, there are no reliable studies confirming the beneficial effect of taking up PA by MS patients on the decrease in the level of kinesiophobia, which would be a good predictor of the effectiveness of rehabilitation. This suggests research in this direction. An argument may be the report on the effectiveness of the 12-week Pilates program in people suffering from lower back pain. They showed a significant reduction in disability (the Roland Morris Disability Questionnaire), pain (VAS), and kinesiophobia (TSK) after 6 weeks from the end of the study [62]. Another study found that a lower level of kinesiophobia before treatment was a predictor of better treatment outcomes in terms of quality of life and level of disability. This report also emphasizes the role of pain neuroscience education (PNE), which, in combination with individual patient physiotherapy aimed at gradual restoration of movements that were previously avoided due to fear of pain, turned out to be effective in reducing the level of kinesiophobia [63].

Acceptance of disease is defined as the process by which patients adapt to changes imposed by the disease. People with a high level of acceptance of the disease generally cope better with the deteriorating health condition and the emerging negative emotions [64]. Research shows that people with a higher level of AIS also show greater confidence in medical personnel and the entire treatment process [33]. In the authors’ own research, a trend was observed that with increasing disability, the level of acceptance of the disease (AIS) decreased. This may be due to the fact that patients from 4 EDSS points begin to feel more acutely the impact of the disease on their daily life and gait function. This indicates the special importance of psychological support at this stage of the disease. One should also remember possible other chronic diseases that affect the well-being and functioning of patients. This aspect was not analyzed in this study, which is one of its limitations. However, this should be taken into account in future research.

The regression results explain the problem of kinesiophobia in 44%. Pain, which was the original motive of the founders of TSK, did not turn out to be statistically significant. This suggests that in MS patients, in addition to functional limitations, other factors, mainly psychological ones, are also important. According to the authors, this is an important discovery changing the importance of determinants of kinesiophobia in patients with MS. Earlier, cited reports of kinesiophobia in people with other diseases were rather associated with acute pain. Chronic pain does not seem to have such a significant effect on kinesiophobia. However, this requires confirmation in further studies. The limitations of this study, especially its cross-sectional nature, the number of people tested, and the limited number of variables studied, and the importance of this problem indicate the need for further research.

## 5. Conclusions

The problem of kinesiophobia is significant in MS patients, and its predictors are the functional status of the patients, low level of acceptance of the disease, and low level of physical activity. The age of the patients and the duration of the disease are not determinants of fear of movement. It should be assumed that the effectiveness of rehabilitation in these patients will require overcoming the fear of movement. It is suggested that doctors, physical therapists, and psychologists should be involved in this process. The problem of kinesiophobia in MS patients and the effectiveness of overcoming it require further research.

## Figures and Tables

**Table 1:** Descriptive statistics of the studied variables and sex comparison.

Variable	Sex	Avg. (SD)	Median	95%CI	*p*
age	female	45.48 (8.50)	43.50	43.29–47.68	nss
	male	45.60 (9.04)	43.00	41.37–49.83	
duration of the SM	female	10.20 (7.53)	8.50	8.26–12.15	nss
	male	9.25 (3.96)	10.00	7.40–11.10	
VAS	female	3.58 (2.36)	4.00	2.97–4.19	nss
	male	3.45 (2.59)	3.00	2.24–4.66	
EDSS	female	3.23 (1.50)	3.00	2.85–3.62	nss
	male	3.85 (1.60)	4.00	2.24–4.66	
AIS	female	30.55 (8.23)	32.00	28.42–32.68	nss
	male	26.60 (9.37)	28.50	22.21–30.99	
TSK	female	36.28 (8.27)	37.50	34.16–38.41	nss
	male	37.75 (8.01)	40.00	34.00–41.50	
PA	female	4.63 (3.24)	3.07	3.79–5.46	nss
	male	6.52 (5.97)	3.53	3.72–9.31	

Abbreviations: nss, not statistically significant; MS, multiple sclerosis; VAS, visual analogue scale; EDSS, Expanded Disability Status Scale; AIS, Acceptance of Illness Scale; TSK, Tampa Scale of Kinesiophobia; PA, physical activity.

**Table 2:** Correlations of the studied variables with the functional state according to EDSS.

Variable		EDSS						
		1	1.5	2	3	4	5	6
		*n* = 7	*n* = 8	*n* = 12	*n* = 13	*n* = 18	*n* = 15	*n* = 7
age	avg.	40.43	41.13	41.92	41.77	49.56	51.33	45.86
	median	42.00	37.50	42.00	39.00	50.50	51.00	44.00
	r−	0.404 ***						
duration of the MS	avg.	5.39	3.63	10.17	10.26	9.50	13.20	15.14
	median	6.00	2.75	9.50	10.00	8.50	12.00	14.00
	r−	0.405 ***						
VAS	avg.	1.57	3.13	1.92	3.23	5.17	4.27	3.71
	median	0.00	3.00	1.50	3.00	5.00	5.00	5.00
	r−	0.365 **						
AIS	avg.	36.00	36.00	33.83	30.77	27.28	25.00	21.86
	median	38.00	39.00	36.00	30.00	27.00	25.00	21.00
	r−	−0.532 ***						
TSK	avg.	29.14	29.63	32.00	36.54	41.11	40.20	41.29
	median	28.00	28.50	30.50	40.00	41.00	40.00	41.00
	r−	0.535 ***						
PA	avg.	8.96	5.07	4.61	7.55	3.38	4.68	2.89
	median	8.73	4.37	2.80	5.87	2.40	3.86	1.70
	r−	−0.274 *						

Abbreviations: * *p* < 0.05; ** *p* < 0.01; *** *p* = 0.000; EDSS, Expanded Disability Status Scale; MS, multiple sclerosis; VAS, visual analogue scale; AIS, Acceptance of Illness Scale; TSK, Tampa Scale of Kinesiophobia; PA, physical activity.

## Data Availability

Data are archived with the first author and in the Department of Neurology of the University Hospital where they were obtained. If necessary, contact the first author by e-mail at dwasiuk@sum.edu.pl or www.katedrafizjoterapii.sum.edu.pl (accessed on 25 January 2022).
